# Dynamic alterations in white blood cell counts and SARS-CoV-2 shedding in saliva: an infection predictor parameter

**DOI:** 10.3389/fmed.2023.1208928

**Published:** 2023-06-15

**Authors:** Parisa Shiri Aghbash, Reyhaneh Rasizadeh, Milad Shirvaliloo, Javid Sadri Nahand, Hossein Bannazadeh Baghi

**Affiliations:** ^1^Immunology Research Center, Tabriz University of Medical Sciences, Tabriz, Iran; ^2^Department of Virology, Faculty of Medicine, Tabriz University of Medical Sciences, Tabriz, Iran; ^3^Infectious and Tropical Diseases Research Center, Tabriz University of Medical Sciences, Tabriz, Iran

**Keywords:** SARS-CoV-2, COVID-19, laboratory tests, biomarkers, blood cell count, sputum, non-sputum

## Abstract

**Introduction:**

The recent coronavirus (COVID-19) outbreak posed a global threat and quickly escalated to a pandemic. However, accurate information on potential relationships between SARS-CoV-2 shedding in body fluids, especially saliva, and white blood cell (WBC) count is limited. In the present study we investigated the potential correlation between alterations in blood cell counts and viral shedding in saliva in a cohort of COVID-19 patients.

**Method:**

In this preliminary clinical research, 24 age-matched COVID-19 patients without comorbidities, 12 (50%) men and 12 (50%) women, were followed up for a period of 5 days to investigate whether changes in the level of viral shedding in saliva might parallel with temporal alterations in WBC count. Viral shedding in saliva was qualitatively measured by performing SARS-CoV-2 rapid antigen tests on patient saliva samples, using SARS-CoV-2 Rapid Antigen Test Kit (Roche, Basel, Switzerland). These patients were classified into two groups with sputum and non-sputum cough. WBCs counts including leukocyte (LYM), neutrophil (NEU), and LYM counts were recorded for each patient on days 1, 3, and 5.

**Results:**

The results of the present study showed that the levels of WBC, LYM, and NEU as well as erythrocyte sedimentation rate (ESR) increased significantly on the 5th day compared to the first day in both groups with sputum. However, the levels of C-reactive protein (CRP), Neutrophil-to-Lymphocyte Ratio (NLR) and lactate dehydrogenase (LDH) did not show significant changes.

**Conclusion:**

This study proves that investigating the change in the number of blood LYMs as well as laboratory parameters such as CRP, LDH, and ESR as biomarkers is an accurate indicator to detect the amount of viral shedding in people with sputum and non-sputum. The results of our study denote that the measured parameters exhibit the intensity of viral shedding in people with sputum.

## 1. Introduction

Severe acute respiratory syndrome (SARS), Middle East respiratory syndrome (MERS), and coronavirus disease 2019 (COVID-19) are all examples of outbreaks that pose a hazard to public health that are caused by coronaviruses, which primarily attack the human respiratory system (1). Severe acute respiratory syndrome coronavirus 2 (SARS-CoV-2) was first identified in patients with atypical pneumonia in the Chinese province of Wuhan in December 2019, and it has spread quickly around the world, causing substantial lung inflammation and acute respiratory distress syndrome (ARDS) (2), particularly in elderly patients with underlying comorbidities (1). Patients with COVID-19 can spread the virus to multiple people during the early subclinical period before being diagnosed. Usually the symptoms are mild in the early stages of the disease, but the patient’s condition rapidly deteriorates 7–10 days after the infection, leading to death in 1.4–2.3% of patients (3). Common symptoms include fever, cough, sore throat, shortness of breath, myalgia, and malaise (4). Myalgia is frequently suggested to be a result of cytokine response and widespread inflammation, and is reported in up to 36% of patients with COVID-19 at the onset of disease, according to many investigations (5). In most cases, severity is mild to moderate and many remain asymptomatic (6).

In this way, evaluation of viral shedding in a variety of bodily fluids, such as saliva, is necessary to comprehend the dynamics of transmission and improve infection control procedures. In addition, numerous variables of the disease, including clinical considerations, patient condition and outcome have been the subject of studies pertaining COVID-19. To assist physicians with the diagnosis, treatment and prognostication of COVID-19 patients, a description of the clinical characteristics associated with COVID-19 is essential (7). It is predicted that in severe COVID-19 patients whose immune responses are dysregulated, hyperinflammation is exacerbated and neutrophils (NEUs) may aggravate pathological damage (8). Neutrophil-to-Lymphocyte Ratio (NLR) is a clinical indicator of balance between the innate immune response and adaptive immunity, and increased NLR is regarded as an early indicator of the severity of COVID-19 (7). Lymphopenia is a significant component of severe COVID-19, and a lymphocyte (LYM) count may be helpful in determining the severity of clinical consequences (9). It is well known that cytotoxic T lymphocytes (CTLs) and natural killer (NK) cells are crucial in the fight against viral infections. Recent studies have shown that a significant portion of severely sick COVID-19 patients, up to 85%, suffer from lymphopenia, which is characterized as an unusually low number of LYMs (10). Despite the possibility of a temporary rise in T cells at the outset of COVID-19, these individuals often had low LYMs counts (10). Recent research indicates that a significant subset of COVID-19 patients may be susceptible to developing cytokine storm syndrome. This hyperinflammatory condition is known to be associated with viral infections and can trigger a potentially life-threatening complication known as secondary hemophagocytic lymph histiocytosis (10). Monitoring of NLR may assist physicians in early detection of high-risk COVID-19 patients as it takes into consideration both NEU and LYM levels, delivering a thorough, accurate, and reliable index for comparative purposes (11). In the context of severe cytopenia, it is advisable to account for NEU and LYM counts, as patients with such conditions may exhibit a heightened likelihood of salivary HSV-1 shedding (12).

Moreover, clinical characteristics and laboratory findings associated with COVID-19 may vary in different age groups (13). Some laboratory tests have also been reported to change in COVID-19 patients, including white blood cells (WBCs), NEU ratio, LYM count, C-reactive protein (CRP), erythrocyte sedimentation rate (ESR) and creatinine (13). CRP, for instance, has been proposed as a useful biomarker for predicting the likelihood of exacerbation in non-severe COVID-19 patients (14). In this study, we aimed to investigate the extent of changes in WBCs such as NEUs and LYMs during the infection, and to see whether changes in the level of viral shedding in saliva might be parallel to temporal alterations in WBC counts.

## 2. Method and material

### 2.1. Study design

This study was conducted on patients with RT-qPCR-confirmed COVID-19 who had referred to tertiary institutional hospital in northwestern Iran. Patients without underlying health conditions who had presented with lymphopenia at the time of admission were deemed as potentially eligible participants. A total of 24 COVID-19 patients who met these criteria were enrolled at the study. We qualitatively measured viral shedding in saliva by performing SARS-CoV-2 rapid antigen tests on patient saliva samples, using SARS-CoV-2 Rapid Antigen Test Kit (Roche, Basel, Switzerland). Participants were classified into two groups, sputum and non-sputum, based on the state of sputum expectoration in cough.

### 2.2. Participants

In this preliminary clinician study, 24 age-matched COVID-19 patients, 12 (50%) men and 12 (50%) women, were followed up for a period of 5 days to investigate whether changes in the level of viral shedding in saliva might be parallel to temporal alterations in WBC counts. Three consecutive series of saliva samples were collected from patients at 6 a.m. on day 1, 3, and 5. The samples were collected under the supervision of an expert and tested for the presence of viral antigens with rapid antigen testing (Table 1).

**Table 1:** Samples collections during 3 periods.

Day 1	Day 3	Day 5
Sample collection, first series	Sample collection, second series	Sample collection, third series
Retrieval of lab test values: WBC, neutrophil, lymphocyte, CRP, ESR (1st hour) and LDH	Retrieval of lab test values: WBC, neutrophil and lymphocyte	Retrieval of lab test values: WBC, neutrophil and lymphocyte
Subjective evaluation of myalgia, perceived by patients as body pain	Subjective evaluation of myalgia, perceived by patients as body pain	Subjective evaluation of myalgia, perceived by patients as body pain

### 2.3. Data gathering

Pharyngeal swab samples were collected for RT-qPCR COVID-19 test on arrival. Blood samples were collected from each participant and routine blood test such as WBC counts including leukocyte, NEU and LYM counts were recorded for each patient on days 1, 3 and 5. By the end of the 5^th^ day, a total of 72 samples had been tested. NLR was calculated by dividing the total number of NEUs by the total number of LYMs from the peripheral blood sample, or the percentage of NEUs by the percentage of LYMs (7). Also, circulating pro-inflammatory markers such as CRP, ESR and lactate dehydrogenase (LDH) were measured as part of the routine COVID-19 in-patient care.

### 2.4. Statistical analysis

Data collected from patients were then imported into an Excel Spreadsheet for statistical analyses. Data on WBC, NEU, LYM, NLR levels were expressed as confidence interval (CI = 95%) and analysis with two-way ANOVA in GraphPad Prism v8. Differences in the levels of CRP, LDH, and ESR between the sputum and non-sputum patients were assessed using Mann–Whitney test in GraphPad Prism v8 (San Diego, California, United States^1^). Receiver operating characteristic (ROC) curve and AUC were used to analyze the optimal cut-off for prediction of sputum and non-sputum patients. In the current study, an AUC of 0.9 to 1 was defined as excellent accuracy, 0.8 to 0.9 as very good, 0.7 to 0.8 as good, 0.6 to 0.7 as sufficient, 0.5 to 0.6 as equivocal, and <0.5 as poor.

## 3. Results

### 3.1. Clinical characteristics of patients

A total of 24 patients, 12 (50%) men and 12 (50%) women, with a mean age of 53.91 ± 2.62 (range: 50–59) were included in this study, of whom 50% were classified as patients with sputum and 50% as non-sputum. Laboratory parameters and clinical characteristics are summarized in Table 2. All our patients had mild to moderate disease. Severe COVID-19 was not considered in this study.

**Table 2:** Patients’ demographic characteristics.

Variable	Sputum			Non-sputum		
Male	6			6		
Female	6			6		
Age	52			55		
Myalgia	Day 1	Day 3	Day 5	Day 1	Day 3	Day 5
	83.3%	41.6%	16.66%	66.6%	33.3%	16.66%
NEU	69.3%	68.4%	64.5%	66.7%	65.7%	66.2%
LYM	13.6%	15.7%	19.4%	12.9%	21.1%	16%

### 3.2. Laboratory parameters

**Figure 1:**
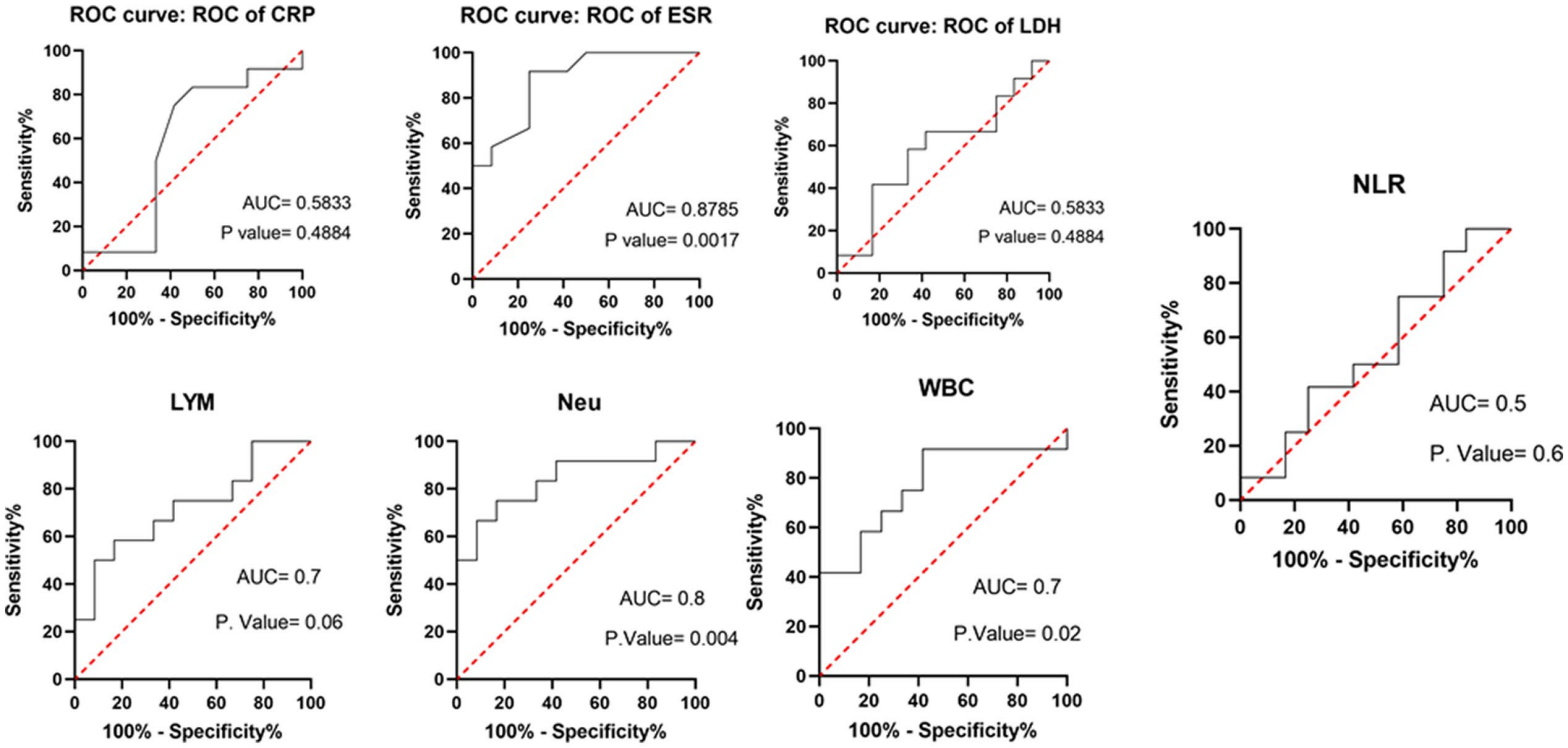
Area under the receiver operating characteristic curve of different laboratory parameters and immune cells (WBC, LYM, Neu) in predicting sputum and non-sputum cases.

**Figure 2:**
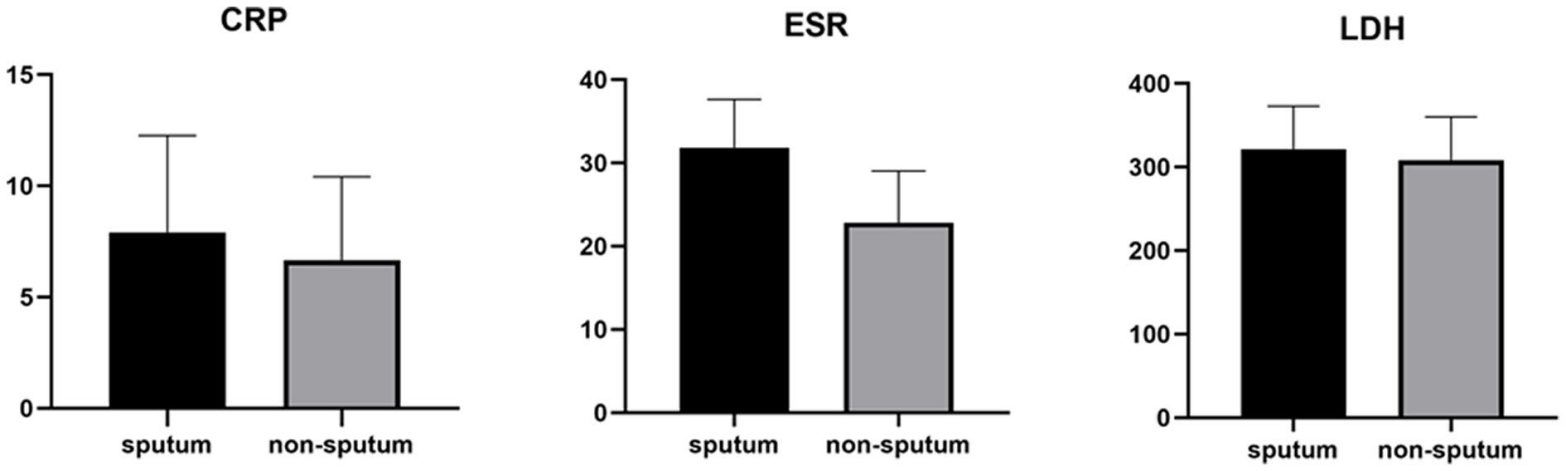
Measurements of LDH, CRP and ESR represented per group. The box plots show the data from all analyzed patients. In every box plot, we show the mean values and the standard error of the means. In every box plot, the upper line is the highest measurement detected. All measurements are grouped as sputum and non-sputum.

**Figure 3:**
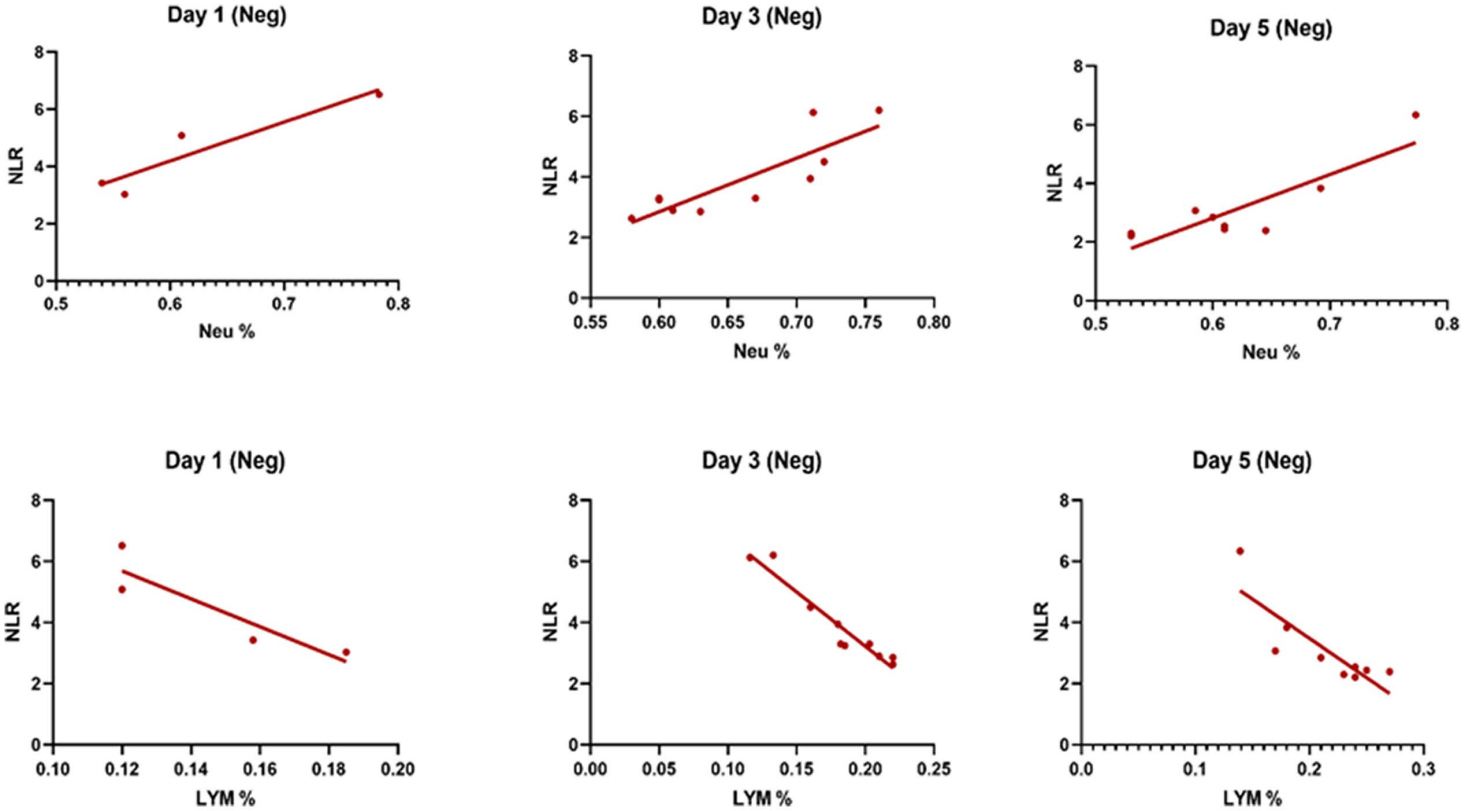
The linear regression analysis of NLR-Neu% and NLR-LYM% of patients in the negative type in 5 days. Within the five-day period after admission Neu-weighted NLR shows an increase, while Lym-weighted NLR exhibits a fall, suggesting a shift from neutrophil-dominant to neutrophil-balanced illness, which was statistically significant (*p* < 0.05).

**Figure 4:**
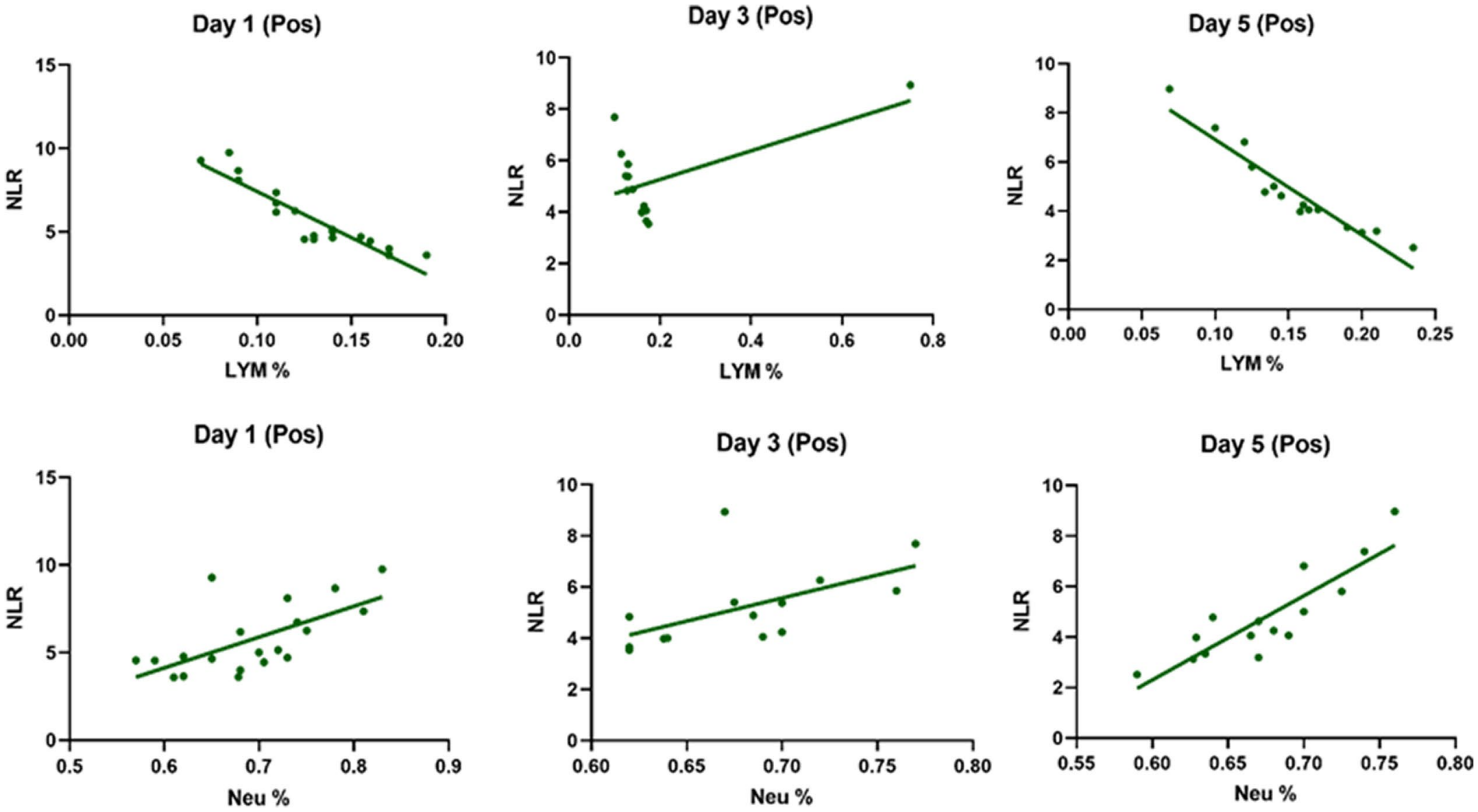
The linear regression analysis of Neu-weighted NLR and Lym-weighted NLR of patients in the positive type in 5 days. The level of NLR in the positive group decreases with the increase in the lymphocytes counts due to increased shedding of virus in saliva, while the amount of NLR promotes significantly with the increase in the number of neutrophils.

Table 3 compares the laboratory parameters of sputum and non-sputum patients. In sputum patients, the levels of LYM, WBC, NEU, and ESR were significantly higher. However, no significant differences were found in CRP, LDH, NLR in sputum patients compared to non-sputum patients. Figure 1 shows the area under the ROC curve (AUC) of CRP, LDH, and ESR for both groups. We found that while CRP (AUC = 0.5833) and LDH (AUC = 0.5833) did not show significant accuracy in predicting viral shedding intensity, ESR demonstrated good accuracy in predicting viral shedding in COVID-19 patients. Moreover, a statistically significant difference was noted in ESR between the groups (Figure 2). In Figures 3, 4, we also analyzed the Neu, LYM, and NLR amounts with regression diagrams. It has been shown that 5 days after admission, Neu-weighted NLR increases while LYM-weighted NLR decreases, indicating a statistically significant change from NEU-dominant to NEU-balanced disease. Moreover, due to increased viral shedding in saliva, the level of NLR in the COVID-19 positive group falls as LYM counts rise, but NLR levels considerably increase as NEU counts rise.

**Table 3:** Comparing the laboratory parameters between the sputum and non-sputum COVID-19 cases.

Parameters	All patients (15)	Sputum (Group 1)	Non-sputum (Group 2)	*p* value
WBC	581.5–1,424	7,246	6,243	<0.0001
Neutrophil	552.6–1,153	4,972	4,119	<0.0001
Lymphocyte	96.97–424.9	1,214	953.1	0.0027
NLR	−0.8581–0.2659	4.634	4.930	0.2915
CRP	−4.692–2.192	8.5	6	0.5029
LDH	−56.96–30.29	317.5	302.5	0.5040
ESR	−14.09 to −3.909	31.50	23.50	0.0009

## 4. Discussion

The public health is now threatened globally by newly developed coronavirus (2). While the majority of patients develop moderate disease, some patients can progress to pneumonia, ARDS, and multi-organ failure (6, 16, 17). It has been confirmed that several laboratory parameters may differ among COVID-19 patients with varying degrees of severity (13, 16). In spite of a competent immune system, hyperinflammation may still occur in COVID-19, resulting in ARDS (8). Pathogen removal by phagocytosis is the main function of NEUs, which are part of the innate immune system. In addition, they show a variety of additional immunological activities, including cytokine production to limit viral replication (8), and secreting leukotrienes and reactive oxygen species (ROS) (18). Based on the findings of this study Neu, LYM, WBC count, NLR, and ESR level have very good accuracy in viral shedding in saliva to temporal alterations in COVID-19 patients. In line with our results, Eun Kim et al. also showed that the viral load kinetics of saliva in patients with sputum resembled those of nasopharyngeal/oropharyngeal swabs; however, the viral load kinetics of saliva in patients without sputum was more comparable to those of gastrointestinal tract samples, including stool, than respiratory samples (19).

In addition, analysis of the laboratory findings of our patients showed that the levels of NEU and LYM in the sputum group increased during five-day period compared to the non-sputum group; while, NLR exhibited a significant decrease in the sputum group compared to the non-sputum group. As a result, according to our findings, Neu can be considered as a predictive factor for viral shedding (or viral clearance) in the early stages of the disease and the amount of sputum secretions. The results of the ROC curve in our study also showed, based on an AUC = 0.8, NEUs can be considered as a type of diagnostic factor with appropriate specificity and sensitivity. In this regard, it has been reported that the number of circulating NEUs increases gradually as COVID-19 progresses, and NEU extracellular traps (NETs), the extracellular network of NEUs that produce DNA/histone proteins to control infection, lead to increase the intensity of inflammation (20). NEUs and LYMs are the primary components of the human defense system against infection (21).

Lymphopenia and leukopenia have been observed in patients with COVID-19, which can be due to increased expression of FAS and FAS-L in T-cells, B-cells, and NK cells and as a result of increased apoptosis in these cells. Also, as a result of the increased expression of PD-1 and TIM-3 on the CD4+ and CD8+ T-cells, it leads to the exhaustion of T cells and deregulation of inflammatory cytokines secretion (22, 23). It has also shown that the WBC level in patients with mild symptoms is higher than its normal values (24). An increase in WBC indicates a promotion in the cellular immune system response to control viral infection and as a result, an increase in the inflammatory cytokines expression such as IL-10 and CRP (15). Moreover, it has been shown that following an increase in viral load, various anti-inflammatory cytokines such as IFN-γ or IL-10 are secreted as a result of an increase in CD4+ or CD8+ T lymphocytes, which lead to a decrease in inflammation and disruption of viral replication. In general, the increase of WBC in COVID-19 patients, regardless of age or sex, can be used as a diagnostic marker in the early stages of the disease (25, 26).

**Figure 5:**
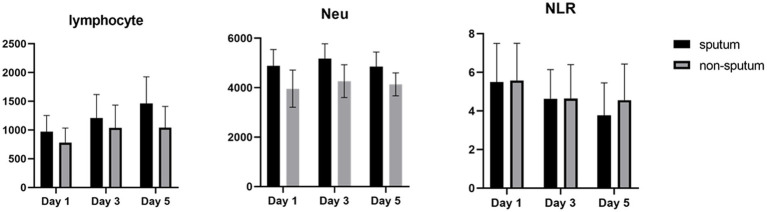
The degree of changes in WBCs and NLR in sputum (black) and non-sputum (gray) groups.

After analyzing the data related to laboratory results, we noticed significant differences between the first and the 5th day following hospitalization of patients (sputum and non-sputum). Accordingly, we examined the changes in the levels of LYMs, NEUs, WBC, and NLR during this period (Figure 5). The correlation between the amount of sputum secretion was analyzed using ROC curves. The NLR AUC was approximately 0.5, which cannot be used as potential diagnostic biomarkers for analysis. Nevertheless, it was observed that the levels of WBC, LYM, and Neu can predict the amount of sputum secretion and the predictive effect of Neu was significant in the sputum group compared to non-sputum during this period. In line with our observations, Feng et al. demonstrated that immune-inflammatory measurements including WBC count, LYM count, PCT, CRP, and NLR may indicate COVID-19 progression. However, in contrast to our results they suggested that monitoring of NLR may assist physicians in early detection of high-risk COVID-19 patients (6). Moreover, according to the findings of Moradi et al., ischemic heart disease (IHD), age, NEU count, and NLR may all be thought of as predictors of survival in COVID-19 patients, which generally corroborate studies from other countries (27).

In contrast, in the current study, the level of NLR decreased during the 5 days of investigation and since the NLR level has a negative correlation with the level of LYMs and positive correlation with NEUs, it was shown that the number of lymphocytes increases as the level of NLR decreases.

According to another study performed by Xia et al., WBC count, NEU count, NLR, platelet to lymphocyte ratio (PLR) and neutrophil-to-platelet ratio (NPR) were all substantially enhanced in the severe groups than in the non-severe group. The LYM count, basophil count, red blood cell (RBC), haemoglobin (HGB), hematocrit (HCT), and lymphocyte-to-monocyte ratio (LMR), on the other hand, were all significantly lower in the group with severe disease. The findings imply that in severe COVID-19 patients, NLR may represent a novel diagnostic biomarker (28). Naoum et al. indicated that it is possible to distinguish between severe and non-severe presentations in COVID-19 patients by using baseline WBC counts and comprehensive cell population data (CPD) measures, as the baseline WBC and NEU counts were higher in COVID-19-positive individuals who were later hospitalized or died (29). Moreover, Li et al. demonstrated a higher count of WBCs, NEUs, LYMs, CRP, fibrinogen, D-dimer, creatine kinase, and LDH levels in severe COVID-19 patients compared to their non-severe counterparts, and strong correlations between CRP and LDH and other indices were observed, which suggested that these two variables had a significant impact on the severity of COVID-19 (30). Almigdad H. M. et al. also reached similar results such as increased CRP and LDH levels in acute COVID-19 patients than negligible patients (31). LDH is a metabolic and immune biomarker, and the immune-suppressive cells such as macrophages and dendritic cells (DCs) are enhanced by LDH; however, cytolytic cells such as NK cells and cytotoxic T-lymphocytes are inhibited. In addition, as shown using cellular studies, in the early stages of SARS-CoV infection, there is secretion of cytokines and chemokines in respiratory epithelial cells, and immune cells such as dendritic cells and macrophages. These cells secrete low levels of INF-γ, and high levels of pro-inflammatory cytokines such as IL-6, IL-1β and TNF-α (32). Han et al. discovered that LDH was negatively connected with LYMs and their subsets, including CD3+, CD4+, and CD8+ T cells, and positively correlated with CRP (33). In our study, also, it was shown that there is no significant difference in CRP and LDH levels between the sputum and non-sputum groups in the patients examined in this study. Besides, the blood level of ESR showed a significant difference between the two groups. The present research demonstrates that the AUC of ESR exceeds 0.8, indicating its suitability and high predictive capacity in forecasting SARS-CoV-2 infection.

Previous research found that lymphopenia is frequently seen in people with SARS-CoV, and has a reported incidence of 69.6–54% (34, 35). SARS infection has been found to elicit two possible pathophysiological outcomes, namely immune-mediated LYM destruction resulting in lymphopenia, or direct inhibition of bone marrow function (36). There exists a possibility of comparable pathogenic mechanisms shared between SARS-CoV-2 and SARS-CoV, such as direct cellular infection and cytokine-mediated LYM destruction (9). Moreover, a meta-analysis suggested that in contrast to individuals with normal or higher LYM counts, patients with COVID-19 who arrived with lower LYM levels upon admission to a healthcare institution showed a greater chance of poor clinical outcomes. Due to COVID-19’s capacity to cause LYM destruction, lymphopenia, a disorder marked by an unusually low number of LYMs in the blood, is frequently noticed in COVID-19 patients. The degree of lymphopenia can offer prognostic data about the course of the illness and the likelihood that a patient will have life-threatening consequences (37).

In addition, it has been shown that LYMs, especially T lymphocytes, may be potentially affected by SARS-CoV-2. In other words, viral particles spread through the respiratory system and by infecting other cells and creating an immune response, they lead to a change in the number of peripheral WBCs such as LYMs (38). In this regard, Tsui et al. showed that due to the high number of NEUs and LDH in the admission of COVID-19 patients, it is a factor for predicting unfavorable clinical outcome (39). So, according to our results and the analysis of the regression curve, the amount of NEU in the positive group is higher than the negative group on the 5th day. Also, in the positive group compared to the negative group, the number of lymphocytes decreases significantly on the 5th day (Figures 3, 4). According to the findings of Pirsalehi et al. study, severe instances of the illness typically show leukocytosis in addition to a statistically significant increase in the mean number of WBCs. This finding implies that a high WBC count may be a possible sign of a poor prognosis (40).

With COVID-19, neurological issues have been reported. Myalgia and headaches are rather typical, although it is uncommon to develop a major neurological condition (41). Liu et al. studied the major early symptoms of the disease which included fever in 81.8%, coughing in 48.2%, and myalgia in 32.1% of the patients, with additional, almost 80% of the patients had normal or reduced WBC counts, and 72.3% had lymphocytopenia (42).

## 5. Limitation

There were some inescapable restrictions on this study. First of all, the sample size was not large. Second, not every patient was constantly observed for all clinical symptoms because this study only focused on blood laboratory measurements. Last but not least, the study was a single-center study. In conclusion, our future research will concentrate on large-sample, multi-center, and systematic prospective studies to address these limitations.

## 6. Conclusion

In general, this study was conducted with the aim of investigating the change in the number of WBCs such as LYM and Neu as well as laboratory parameters such as CRP, LDH, and ESR as biomarkers to detect the amount of viral shedding in people with sputum and without sputum. The results indicate that these parameters, along with WBC counts, correlate with the intensity of viral shedding in individuals with sputum. Moreover, ESR demonstrated promising predictive value, while NEU showed potential as a diagnostic factor with appropriate specificity and sensitivity. These findings suggest that these parameters could be valuable in identifying individuals with viral shedding.

## Data availability statement

The original contributions presented in the study are included in the article/supplementary material, further inquiries can be directed to the corresponding author.

## Ethics statement

The studies involving human participants were reviewed and approved by Tabriz University of medical sciences and under the ethical approval code of IR.TBZMED.REC.1400.326. Written informed consent for participation was not required for this study in accordance with the national legislation and the institutional requirements.

## Author contributions

HB: conceived the idea for this manuscript and edited subsequent drafts. PA: literature search, statistical analysis, and manuscript preparation. RR: literature search, manuscript preparation, and design of the tables. MS: review of the manuscript, manuscript preparation, and sample collection. JN: review of the manuscript. All authors contributed to the article and approved the submitted version.

## Funding

This study was financially supported by a grant from Tabriz University of Medical Sciences (IR.TBZMED.REC.1400.326). This project was supported by the Infectious and Tropical Diseases Research Center, Tabriz University of Medical Sciences, Tabriz, Iran.

## Conflict of interest

The authors declare that the research was conducted in the absence of any commercial or financial relationships that could be construed as a potential conflict of interest.

## Publisher’s note

All claims expressed in this article are solely those of the authors and do not necessarily represent those of their affiliated organizations, or those of the publisher, the editors and the reviewers. Any product that may be evaluated in this article, or claim that may be made by its manufacturer, is not guaranteed or endorsed by the publisher.

## Footnotes

^1^ http://www.graphpad.com
